# Interactions between CD44 and Hyaluronan in Leukocyte Trafficking

**DOI:** 10.3389/fimmu.2015.00068

**Published:** 2015-02-17

**Authors:** Braedon McDonald, Paul Kubes

**Affiliations:** ^1^Department of Medicine, University of British Columbia, Vancouver, BC, Canada; ^2^Snyder Institute for Chronic Diseases, University of Calgary, Calgary, AB, Canada

**Keywords:** inflammation, leukocyte recruitment, leukocyte trafficking, CD44, hyaluronan

## Abstract

Recruitment of leukocytes from the bloodstream to inflamed tissues requires a carefully regulated cascade of binding interactions between adhesion molecules on leukocytes and endothelial cells. Adhesive interactions between CD44 and hyaluronan (HA) have been implicated in the regulation of immune cell trafficking within various tissues. In this review, the biology of CD44–HA interactions in cell trafficking is summarized, with special attention to neutrophil recruitment within the liver microcirculation. We describe the molecular mechanisms that regulate adhesion between neutrophil CD44 and endothelial HA, including recent evidence implicating serum-derived hyaluronan-associated protein as an important co-factor in the binding of HA to CD44 under flow conditions. CD44–HA-mediated neutrophil recruitment has been shown to contribute to innate immune responses to invading microbes, as well as to the pathogenesis of many inflammatory diseases, including various liver pathologies. As a result, blockade of neutrophil recruitment by targeting CD44–HA interactions has proven beneficial as an anti-inflammatory treatment strategy in a number of animal models of inflammatory diseases.

## Introduction

The transit of leukocytes from the bloodstream into inflamed tissues involves a series of sequential events, each mediated by the engagement of adhesion molecules on leukocytes and their ligands on vascular endothelium. First, cells flowing in the bloodstream tether to the vessel wall, and begin rolling along the luminal surface. Recent advances in live-cell imaging have revealed that rolling cells form surface appendages called “long tethers” and “slings” that stabilize the rolling phase to enable this dynamic cell–cell interaction to occur at high shear stress (1, 2). Subsequently, cells firmly adhere to the vascular endothelium, and begin crawling along the vessel wall until they arrive at a favorable site for trans-endothelial migration out of the vasculature (3). Upon arrival within the inflamed tissue, leukocytes are guided by gradients of chemoattractants to shepherd them toward their final target where they carry out their effector functions (4).

Each phase of the multi-step cascade of leukocyte recruitment involves binding interactions between adhesion molecules on leukocytes and their ligands on endothelium. Selectins and selectin ligands are the prototypic adhesion molecules that mediate tethering and rolling, through their ability to form transient adhesive “catch” bonds at high shear stress (5). Firm adhesion is classically ascribed to binding between integrins on immune cells (Mac-1, LFA-1, VLA-4, etc.) and receptors of the immunoglobulin-superfamily on endothelium (ICAM-1, VCAM-1, etc.). The ability of leukocytes such as neutrophils to locomote across the luminal surface of blood vessels via intra-luminal crawling has also been shown to involve integrins, while transmigration requires the coordinated interplay of multiple molecular binding partners (6).

An explosion of research in the field of leukocyte trafficking in the past 25 years has identified many additional “non-classical” adhesion molecules that can support cell recruitment in the myriad of cell, organ, and disease-specific contexts that are required for a functional immune system (7). Among these, CD44 and its ligand, hyaluronan (HA), have emerged as important adhesion molecules required for cell trafficking in multiple organs, and contribute to the pathogenesis of a variety of inflammatory diseases. CD44 is a type I transmembrane glycoprotein expressed by multiple hematopoietic and non-hematopoietic cells. CD44 is encoded by a single gene, but can be expressed in over 20 isoforms as a result of alternative splicing, and can undergo an array of post-translational modifications, enabling nuanced regulation and diverse functions. The key ligand for CD44, HA, is a member of the glycosaminoglycan family of extracellular matrix molecules, formed from a basic structural unit of repeating dissacharides of *N*-acetylglucosamine and *N*-glucuronic acid. HA is a nearly ubiquitous component of the extracellular matrix, and can be produced by multiple cell types, including endothelium [for a comprehensive review of the regulation of HA synthesis and metabolism, see Ref. (8)]. Below, we review the biology of CD44–HA interactions in immune cell trafficking, explore the molecular mechanisms that regulate the binding between these molecules, and highlight the critical role of CD44–HA-mediated leukocyte recruitment in the pathogenesis of inflammatory diseases.

## CD44 and Hyaluronan in the Leukocyte Recruitment Cascade

### Rolling

Seminal experiments using a parallel-plate flow-chamber system to simulate the hemodynamic environment of post-capillary venules were the first to demonstrate a role for CD44 and HA in leukocyte trafficking. Lymphocytes were observed to roll on HA-coated plates, and this rolling could be inhibited by incubating the lymphocytes with a function-blocking antibody against CD44 (9). Subsequently, similar *in vitro* systems were used to demonstrate that CD44 on lymphocytes could support rolling on a monolayer of cultured endothelial cells in an HA-dependent manner (9, 10). These findings were confirmed *in vivo* in a model of peritonitis, in which T-cells utilized CD44–HA interactions to home to the inflamed peritoneal cavity (10). Recruitment of superantigen-activated T-cells into the inflamed peritoneum could be inhibited by CD44-blocking antibodies, as well as intravenous infusion of hyaluronidase to degrade endothelial HA (10). However, unlike lymphocytes, neutrophils are unable to roll on a purified HA substrate *in vitro*, and neutrophil rolling on cultured endothelial cells as well as the endothelium of post-capillary venules *in vivo* is not dependent on CD44–HA interactions.

In addition, CD44 can also contribute to leukocyte rolling through interactions with non-HA ligands. Teder and colleagues identified CD44 as a novel E-selectin ligand, and showed that binding between these molecules can support neutrophil rolling in mice and humans (11). Specifically, engagement between neutrophil CD44 and endothelial E-selectin was required for slow rolling, whereas P-selectin-PSGL-1 interactions were required for initial tethering and fast rolling. Furthermore, neutrophil CD44 from patients with leukocyte adhesion deficiency (LAD) syndrome type II (due to mutations of the GDP-fucose transporter gene, resulting in complete deficiency of fucosylated selectin-ligand moieties) was unable to bind E-selectin, implicating CD44–E-selectin interactions in the pathogenesis of this disease (11). In addition to neutrophils, T-lymphocytes also utilize CD44–E-selectin interactions for slow rolling *in vivo*, in a manner that is independent of the contribution of CD44–HA interactions (12).

### Firm adhesion

Intravital microscopy of the inflamed cremaster muscle of mice has revealed that unlike rolling, firm adhesion of neutrophils can be supported by interactions between CD44 and HA. Blocking antibodies against CD44, genetic deletion of CD44, and removal of HA from the vascular endothelium have each been shown to attenuate neutrophil adhesion (but not rolling), within post-capillary venules (13). Similarly, as described below, CD44 can support neutrophil adhesion within low-shear microvascular beds such as the liver sinusoids, in which neutrophils undergo primary adhesion without a pre-requisite rolling step (14).

A role for CD44 in mediating firm adhesion of lymphocytes has been suggested, but the exact functional contribution of CD44 is less clear. Both *in vitro* and *in vivo*, disruption of CD44–HA interactions limits the adhesion of activated lymphocytes to endothelium (10, 15). However, given that CD44–HA binding also mediates the pre-requisite rolling step, the reduction of firm adhesion may be a secondary effect. This question has been addressed directly by Nandi and colleagues, who investigated the contribution of CD44 versus integrins (the prototypic molecules supporting firm adhesion) to adhesion of activated T-cells to endothelium under flow conditions (16). Interestingly, it was found that CD44 was required for firm adhesion, although not through binding interactions with HA. Co-immunoprecipitation experiments revealed bi-molecular complex formation between CD44 (which supports rolling) and the integrin VLA-4 (which supports adhesion) (16). Expression of truncated forms of CD44 that lacked the cytoplasmic tail prevented physical coupling of CD44 with VLA-4, resulting in cells that could roll (using CD44) but were unable to adhere. These experiments revealed that while CD44–HA interactions support lymphocyte rolling, CD44 is also required for adhesion by directly collaborating with integrins to enable the formation of high affinity binding to VCAM-1.

### Trans-endothelial migration and chemotaxis

Conflicting data exist regarding the function of CD44 and HA in trans-endothelial migration and chemotaxis through interstitial tissues. With respect to transmigration, neutrophil transit across epithelial monolayers *in vitro* was found to be attenuated when neutrophil CD44 was activated, but was unaffected by functional blockade of the HA-binding domain (17). Importantly, these experiments tested neutrophil migration across intestinal epithelial monolayers under static conditions, and therefore their applicability to trans-endothelial migration under flow is unknown. In contrast, visualization of neutrophil recruitment *in vivo* within the inflamed cremaster muscle of mice demonstrated reduced transmigration in CD44 knockout animals (13). The molecular mechanism by which CD44 contributes to trans-endothelial migration has not been studied. Similarly, the role of CD44–HA interaction in chemotaxis is incompletely understood. Khan et al. found conflicting results between the ability of CD44-deficient neutrophils to migrate toward chemoattractants *in vivo* versus *in vitro* (13). In an under-agarose migration assay, neutrophils from CD44-deficient mice had markedly impaired migration toward MIP-2/CXCL2 compared to wild-type neutrophils. However, neutrophil chemotaxis within cremaster muscle *in vivo* was independent of CD44–HA interactions. The differences observed between *in vitro* and *in vivo* conditions is similar to that observed for the role of integrins in neutrophil chemotaxis, and may be the result of differences in the matrix composition through which cells must migrate in the various models (18, 19). Overall, these studies reveal a paucity of data on the contribution of CD44–HA interactions to the final phases of leukocyte recruitment, and highlight a need for further research in this area.

## CD44–HA-Mediated Leukocyte Recruitment in Inflammatory Disease

Numerous studies have confirmed the importance of CD44 and HA in leukocyte recruitment within a variety of organs *in vivo*. Antibody blockade of CD44, CD44 deficiency, or enzymatic depletion of endothelial HA (by administration of hyaluronidase) have been shown to decrease neutrophil, monocyte, and/or lymphocyte recruitment and attenuate disease activity in models of arthritis (20, 21), dermatitis (22), peritonitis (10), myositis (13), experimental autoimmune encephalomyelitis (23), orchitis (24), retinitis (25), allergic asthma (26), and graft-versus-host disease (27). However, perhaps one of the best-characterized roles for CD44–HA interactions in pathological leukocyte recruitment is seen in inflammatory liver disease.

The liver vasculature represents a unique structural and hemodynamic environment for leukocyte recruitment, as the majority of infiltrating cells are recruited within the dense labyrinth of low-flow, low-shear sinusoids. As a result, the mechanisms of leukocyte trafficking within these vessels deviate from the classic multi-step paradigm, in that recruitment does not require a rolling phase (28). Instead, cells are observed to undergo primary adhesion within these low-flow vessels. Therefore, the pleiotropic abilities of CD44–HA interactions to support all steps from tethering to firm adhesion make this binding interaction well suited to the biology of the liver sinusoids. Furthermore, multiple studies using a variety of imaging techniques have demonstrated that the luminal surface of liver sinusoidal endothelium is densely coated with HA (14, 29, 30). Unlike other vascular beds, in which endothelial cell CD44 is required to anchor HA to the vessel wall, liver sinusoidal endothelial cells (LSEC) do not use CD44 to anchor HA, but instead express a variety of scavenger receptors that capture circulating HA on the cell surface, and present it to passing leukocytes before finally promoting its endocytosis and clearance from the bloodstream (31–33). However, no studies to date have investigated the functional role of these scavenger receptors in leukocyte adhesion to HA within liver sinusoids.

A prominent role for CD44–HA interactions in immune cell recruitment to the liver was first observed in a model of acute hepatic inflammation due to sepsis/endotoxemia. Direct visualization of neutrophil trafficking within liver sinusoids revealed that blockade of CD44–HA interactions led to a 50–70% reduction in the number of adherent neutrophils (14). While CD44 can engage with other ligands to support leukocyte–endothelial interactions within other vascular beds (E-selectin and VLA-4, as described above), this promiscuous activity is not observed in liver sinusoids, as multiple studies have confirmed that selectins and α4-integrins do not contribute to neutrophil recruitment within the liver (14, 28, 34). Menezes and colleagues performed a more in-depth functional analysis of CD44–HA interaction using confocal intravital microscopy in mouse models of endotoxemia and Gram-negative bacterial sepsis (35). It was found that in addition to mediating initial adhesion, CD44 was also required for subsequent stages of recruitment including cell spreading and the initiation of intravascular crawling.

Subsequently, the role of CD44–HA interactions in cell recruitment to the liver has been observed for other cell types in a variety of inflammatory contexts. First, Shi et al. revealed that monocyte recruitment to foci of *Listeria monocytogenes* infection in the liver was reduced by more than 50% by anti-CD44 antibody treatment (36). Interestingly, this study observed a roughly equivalent reduction in monocyte infiltration when Mac-1–ICAM-1 interactions were blocked, but it is not known how each receptor–ligand pair contributes to cell recruitment. One possibility is that CD44 and Mac-1 function cooperatively to promote initial monocyte adhesion. Alternatively, these molecules may function in a sequential manner, with CD44 mediating initial tethering and adhesion, while Mac-1 supports subsequent intravascular crawling toward the bacterial targets. Similar to monocytes and neutrophils, an important role for CD44–HA interactions has also been observed in cytotoxic T-lymphocyte (CTL) recruitment to the inflamed liver in a mouse model of viral hepatitis (37). Hepatitis B transgenic mice develop substantial lymphocytic inflammatory infiltrates within the liver, whereas transgenic mice that are also CD44 deficient have markedly attenuated CTL recruitment. Interestingly, adoptive transfer experiments revealed that CD44 was required on endothelium, but not on CTLs. This stands in stark contrast to the role of CD44 in neutrophil and monocyte recruitment, in which CD44 is required on leukocytes but not endothelium (14, 36). The mechanism underlying this observation is not known, but possible explanations include interactions with non-HA ligands on CTLs, or possibly a role for CD44 in upstream inflammatory signaling rather than as an adhesion molecule in CTL trafficking.

A number of recent studies have described a critical role for CD44–HA interactions in the pathogenesis of fatty-liver disease, the leading cause of chronic liver disease in United States and a growing problem worldwide (38). CD44-deficient mice have markedly attenuated hepatitis in a mouse model of non-alcoholic steatohepatitis (NASH), induced by administration of a lithogenic diet (39, 40). Leukocytes harvested from lithogenic diet-fed mice display significant upregulation of their HA-binding capacity (39). This correlated with diminished leukocyte infiltration into the livers of CD44-deficient animals. Using a similar model of lithogenic diet-induced hepatic steatosis, Kang et al. observed that CD44-deficient mice displayed reduced leukocyte infiltration into the liver, in addition to broader defects in steatosis development, adipose tissue inflammation, and insulin resistance (40). Therefore, in addition to supporting leukocyte infiltration into the liver and the development of NASH, CD44 may be a keystone to the systemic pathogenesis of obesity-associated diseases and the metabolic syndrome.

Lastly, CD44–HA interactions have been implicated in the trafficking of hematopoietic stem cells (HSC) to the liver. *In vitro* experiments studying adhesive interactions between HSC and cultured LSEC found that binding was partially dependent on CD44–HA interactions (41). Similarly, CD44 blockade reduced adhesion of HSC to frozen liver sections from patients with primary biliary cirrhosis and alcoholic liver disease (41). While these findings await confirmation *in vivo*, a similar role for CD44–HA interactions has been observed in mesenchymal stem cell recruitment to the kidney in an animal model of acute tubular necrosis (42). Together, these findings suggest that CD44–HA interaction are important not only for the generation of inflammatory responses, but may also be critical to the resolution phase and tissue regeneration by facilitating stem cell homing to injured tissues. This would add an additional role to the growing list of contributions made by CD44 to the resolution of inflammatory responses (43).

## Regulation of CD44–HA Interactions in Cell Trafficking

Despite the fact that CD44 is constitutively expressed on most leukocytes, and HA is a ubiquitous component of the extracellular matrix, trafficking leukocytes only adhere to HA under inflammatory conditions (44). Engagement between CD44 and HA can be regulated at a number of levels including the quantity of surface expression, as well as their functional states of activation.

### Regulation of CD44 function

CD44 expression on the surface of leukocytes can be upregulated in response to a variety of stimuli including antigen receptor cross-linking, mitogens, cytokines, chemokines, and bacterial products (44). Numerous studies have demonstrated that upregulation of CD44 expression can lead to augmented recruitment of various leukocyte subsets to sites of inflammation. Conversely, defects that result in impaired CD44 surface expression can result in attenuation of leukocyte trafficking kinetics. For example, neutrophils from Rab27a-deficient mice display reduced surface expression of CD44, and as such were found to have impaired adhesion within liver sinusoids in response to endotoxemia (45). Although the precise pathway through which Rab27a controls CD44 expression is not fully elucidated, *in vitro* observations suggest that this molecule may regulate the intracellular trafficking and surface presentation of CD44 on the plasma membrane within neutrophils. Interestingly, genetic defects in the *Rab27a* gene in humans results in a rare immunodeficiency syndrome (Griscelli syndrome 2) characterized by defective neutrophil, NK cell, and CTL function (46). Given the defects in CD44 expression on neutrophils observed in Rab27a-deficient mice, it will be of interest in future studies to determine if the impairments in CTLs and NK cells are also linked to defective CD44 expression and function.

It is not only the absolute quantity of CD44 expression on the cell surface that regulates binding to HA but also the relative expression compared to other adhesion molecules. Studies of neutrophil adhesion in the liver have revealed that the balance between CD44 and integrin (Mac-1) expression can dramatically alter the role of CD44 in leukocyte recruitment. Menezes and colleagues found that CD44–HA interactions are the dominant mechanisms of adhesion in response to endotoxemia/sepsis, whereas CD44 is dispensable when hepatic inflammation was induced by a single neutrophil chemoattractant (fMLP), wherein adhesion required Mac-1 (35). Analysis of surface expression levels revealed equivalent quantities of CD44 expression regardless of the stimulus, whereas Mac-1 expression was significantly reduced during endotoxemia as a consequence of high circulating IL-10. The authors postulated that during endotoxemia, downregulation of Mac-1 allows for CD44 to dominate, resulting in preferential adhesion via CD44–HA interactions, highlighting the functional importance of relative CD44 expression levels in the control of neutrophil trafficking.

In addition to regulation of surface expression levels, activation of leukocytes can also induce a multitude of post-translational modifications (phosphorylation, glycosylation, sulfation, and others) that yield a structurally activated, HA-avid form of CD44 [reviewed by Puré and Cuff (44)]. The functional significance of such post-translational modifications has been demonstrated *in vitro* using flow-chamber experiments, in which lymphocyte activation, and the resultant modifications of CD44, are required to enable rolling and adhesion upon HA-coated coverslips (47). Similarly, activated lymphocytes that are recovered from inflamed tissues *in vivo* posses functionally activated forms of CD44, and demonstrate enhanced binding to HA compared to naïve lymphocytes (10, 15). Perhaps the most direct evidence for conformational activation of CD44 in the control of CD44–HA interaction comes from studies using the IRAWB14.4 antibody, which binds CD44 and alters its conformation directly to generate high HA-avidity. As a result, this antibody commonly serves as a positive control in HA-binding experiments (14, 15).

### Regulation of HA function

While expression and activation of leukocyte CD44 are clearly important for the regulation of cell trafficking, there is emerging evidence that leukocyte recruitment is also modulated by changes in HA expression and/or function at the level of vascular endothelium. First, endothelial cells have the ability to augment their surface expression of HA under inflammatory conditions. Using cultured endothelial cell lines as well as primary endothelial cells, Siegelman and colleagues found that stimulation with TNFα, IL-1β, or LPS resulted in upregulated surface expression of HA (48). Interestingly, this phenotype was only observed in endothelial cells derived from microvascular beds (but not larger vessels) in which the majority of leukocyte recruitment occurs (48). Using a parallel-plate flow-chamber assay, the authors confirmed that CD44-dependent lymphocyte rolling was increased fourfold upon activated endothelial monolayers as a result of upregulated surface expression of HA (48).

In addition to quantitative regulation of HA expression, leukocyte trafficking can also be modulated by functional changes to the structure of HA polymers. First, HA polymer length has received a great deal of attention in recent years as an important rheostatat for the inflammatory response, as a result of its multi-functional inflammatory/anti-inflammatory signaling properties mediated by low versus high molecular weight HA (49). Polymer length may also regulate the function of HA as an adhesion molecule, as evidenced by the fact that digestion with hyaluronidase impairs leukocyte–endothelial interactions. In addition, a growing body of research suggests that a major regulator of HA–CD44 interactions involves structural modification of HA induced by a variety of HA-binding proteins. Serum-derived hyaluronan-associated proteins (SHAPs), is an HA-binding protein that has been shown to modulate leukocyte trafficking through functional alterations of the adhesion properties of HA. Structurally, SHAP is composed of the heavy chains (HC) of inter-α-trypsin inhibitor, a circulating proteoglycan consisting of a single chondroitin sulfate molecule bound to bikunin (a serine protease inhibitor) and the aforementioned HC (HC1, HC2, HC3) (50). At sites of inflammation, IαI HC (SHAP) are transferred onto HA through a trans-esterification reaction catalyzed by tumor necrosis factor-stimulated gene 6 (TSG-6), yielding a covalently bound SHAP–HA complex (50). SHAP–HA complexes purified from the synovial fluid of patients with rheumatoid arthritis bind with greater avidity to lymphocyte CD44 than native HA alone (51). Furthermore, lymphocyte rolling and adhesion *in vitro* upon a substratum of SHAP–HA is increased more than fourfold compared to HA alone (51). Some studies have suggested that SHAP–HA complexes may enable adhesion regardless of the activation state of leukocyte CD44 (52). Systematic inhibition studies have confirmed that adhesion to SHAP–HA still occurs exclusively between HA and CD44, and that the role of SHAP is indirect through functional alterations of HA macromolecular structure (51). Immunofluorescence imaging has revealed that binding of SHAP induces profound architectural changes to the macro-structure of HA polymers, resulting in coalescence of HA polymers into multi-cell-length cables (52). This concentration of HA polymers may augment binding to leukocytes by enabling high avidity interactions with leukocyte CD44, potentially providing favorable clustering of CD44 within plasma membrane microdomains. Alternatively, formation of SHAP–HA complexes may protect HA from degradation or endocytosis, thereby stabilizing the surface landscape of the vascular endothelium to promote interactions with passing leukocytes.

Serum-derived hyaluronan-associated protein–HA complexes are observed in multiple inflamed organs. High concentrations have been demonstrated in the synovial fluid of patients with rheumatoid arthritis (51), and colon tissue from patients with active inflammatory bowel disease (52). Within the colon of patients with IBD, SHAP–HA complexes were observed primarily around blood vessels, as well as within the hyperplastic muscularis layer (52). Within the liver microvasculature, neutrophil adhesion in response to bacterial LPS is preceded by marked induction of SHAP–HA complex formation on the endothelial surface (14, 53). Unlike HA, which is present in liver sinusoids constitutively at high levels, SHAP–HA complexes are only seen after exposure to bacterial products or other stimuli, and CD44-bearing neutrophils adhere within regions that are richly decorated with SHAP–HA complexes (14, 54). Experiments in mice that lack the SHAP precursor (IαI), and are therefore SHAP-deficient, have revealed that neutrophil adhesion is reduced by ~50% compared to wild-type animals (53). Together, these studies implicate functional “activation” of HA in response to SHAP (and perhaps other HA-binding proteins) in the regulation of leukocyte adhesion. Further studies are needed to clarify the mechanisms responsible for the enhanced avidity of HA for CD44 following binding by SHAP, and whether the macro-structural alteration (such as cable formation) that are seen *in vitro* also occur within the vasculature *in vivo*.

## Conclusion

As illustrated in this issue of *Frontiers*, CD44 and HA contribute to a remarkably diverse spectrum of biologic processes. As leukocyte adhesion molecules, CD44 and HA display a variety of functional roles in cell trafficking, and contribute broadly to the initiation, propagation, and resolution of inflammatory responses. Within many domains, CD44 and HA are being investigated as potential therapeutic targets to modulate disease pathology, and leukocyte recruitment is no exception. Through our expanded knowledge of the mechanisms regulating CD44 and HA interactions, and how these influence inflammatory responses, we will gain further insight into potential therapeutic targets to treat inflammatory diseases.

## Conflict of Interest Statement

The authors declare that the research was conducted in the absence of any commercial or financial relationships that could be construed as a potential conflict of interest.
